# Dynamic Ultrasound Imaging of Extensor Pollicis Brevis Hypertrophy in Proximal Intersection Syndrome: A Case Report and Literature Review

**DOI:** 10.1002/jcu.70018

**Published:** 2025-08-11

**Authors:** Daoukas Stavros, Galanis Dimitrios, Mathew Benoy, Malliaropoulos Nikos

**Affiliations:** ^1^ College of Health and Life Sciences London South Bank University London UK; ^2^ Radiology Department, Queen's Hospital NHS Trust, Barking Havering and Redbridge University Hospital (BHRUT) London UK; ^3^ 12th Orthopaedic Department Metropolitan General Hospital Athens Greece; ^4^ Guys and St. Thomas Hospital London UK; ^5^ Centre for Sports and Exercise Medicine, William Harvey Research Institute Queen Mary University London London UK; ^6^ Rheumatology Department, Mile End Hospital Barts Health NHS Trust London UK

**Keywords:** differential diagnosis, overuse syndrome, sonography, tendinopathy, wrist pain

## Abstract

This case report presents an atypical presentation of proximal intersection syndrome and provides ultrasound‐based evidence of extensor pollicis brevis muscle belly hypertrophy with associated edema in a non‐athletic population. While occupationally induced PIS has been previously documented, the patient's symptoms in this case were linked to repetitive lifting tasks involving prolonged extension of the first metacarpophalangeal joint with a wide grip. Dynamic ultrasound assessment revealed radial displacement of the second compartment tendons by the extensor pollicis brevis muscle during the aggravating movement. This finding suggests a novel pathomechanism, potentially driven by mechanical friction and stress between the first and second dorsal compartments, warranting further investigation.

## Graphical abstract

Dynamic ultrasound revealed extensor pollicis brevis hypertrophy displacing second compartment tendons, suggesting a novel friction‐based mechanism for proximal intersection syndrome. This case highlights the role of dynamic sonography in identifying motion‐dependent tendon interactions underlying overuse‐related wrist pain.

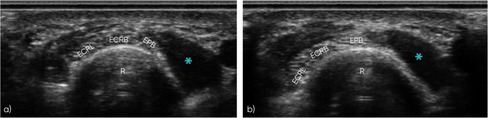

## 1 Introduction

Proximal intersection syndrome (PIS) is characterized by stenosing tenosynovitis at the musculotendinous junctions where the first compartment, comprising the abductor pollicis longus (APL) and extensor pollicis brevis (EPB), intersects with the second compartment, which includes the extensor carpi radialis longus (ECRL), and extensor carpi radialis brevis (ECRB) (Tan et al. 2024; Schmidt et al. 2021; Hoy et al. 2019; Balakatounis et al. 2017). This condition is typically triggered by repetitive wrist motions, leading to friction and inflammation at these crossover points, predominantly 4–6 cm proximal to the Lister's tubercle. Common signs and symptoms include pain, swelling, erythema, and crepitus along the distal dorsal‐radial forearm (Tan et al. 2024; Schmidt et al. 2021; Montechiarello et al. 2010).

This case report explores an atypical presentation of PIS in a non‐athletic individual, where ultrasound findings revealed muscle belly hypertrophy of the EPB and associated edema as potential contributors to the pathology. This report, building on previous occupational presentations of PIS, highlights an alternative pathomechanism based on dynamic sonographic findings and proposes novel risk factors and diagnostic considerations for this condition.

## 2 Case Report

Written informed consent was obtained from the patient for the publication of this case report and accompanying images.

### 2.1 Patient Presentation

A 34‐year‐old female presented at our clinic with symptoms predominantly involving the left dorsal radial aspect of the distal forearm. She is right‐hand dominant but typically uses her left hand for lifting.

**FIGURE 1:**
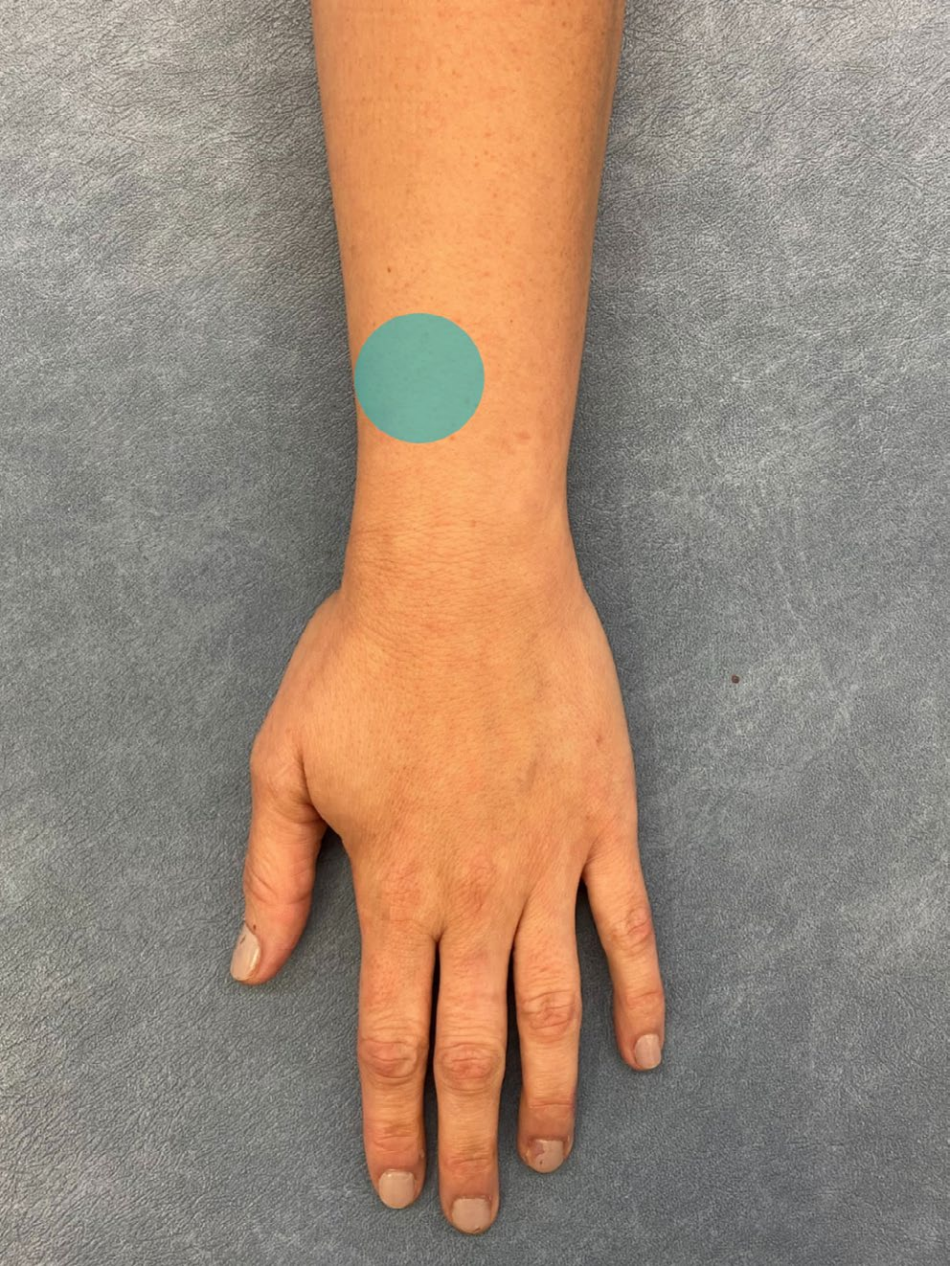
Site of symptoms. The marked area (blue circle) highlights the location of patient‐reported radial‐sided clicking, crepitus sensations, and pain, approximately 4 cm proximal to the Lister's tubercle on the dorsal aspect of the left forearm.

She commenced employment as a full‐time nursery practitioner, a role that involves frequent lifting and holding of small infants and objects. Approximately 5 weeks following her employment initiation, the patient reported a gradual onset of radial‐sided clicking and crepitus sensations on the dorsal aspect of the left forearm, accompanied by pain located approximately 4 cm proximal to the Lister's tubercle (Figure 1). While wrist flexion and extension did not exacerbate symptoms, lifting and holding heavy objects or infants for less than a minute intensified her discomfort. Notably, this pain was cumulative, as repeated lifting throughout her shift resulted in increasing discomfort, even with shorter durations of holding such objects.

The patient reported that the symptoms had developed gradually over a few days to a week, without any recollection of an acute onset or specific trauma. The patient did not observe a particular pattern of symptoms or variations during the day; however, she noted that after days involving more lifting or prolonged periods of holding, the pain worsened at night but would ease off by the next morning. Specifically, she stated that her discomfort was significantly worsened by a specific repetitive activity during her shifts, which involved reaching overhead on tiptoes to lift and lower a 4–5 kg basket with a wide handgrip from the back of a high shelf. She also observed that her pain would generally improve over the weekends when she was not on duty. Despite these symptoms, she was mostly able to perform everyday living activities, albeit with discomfort. However, there were times when the condition significantly affected her ability to carry out daily tasks. Initially, she tried using ice, which provided short‐term relief.

She reported no participation in any sports‐related or recreational activities in the last 12 months. However, she was also involved in household tasks, which included activities that could contribute to repetitive strain, such as lifting and carrying objects during daily chores.

The patient denied any neurogenic symptoms or a history of systemic inflammatory diseases. Additionally, there was no notable medical or surgical history related to the affected limb, nor any history of acute trauma or previous injuries.

### 2.2 Physical Examination

**FIGURE 2:**
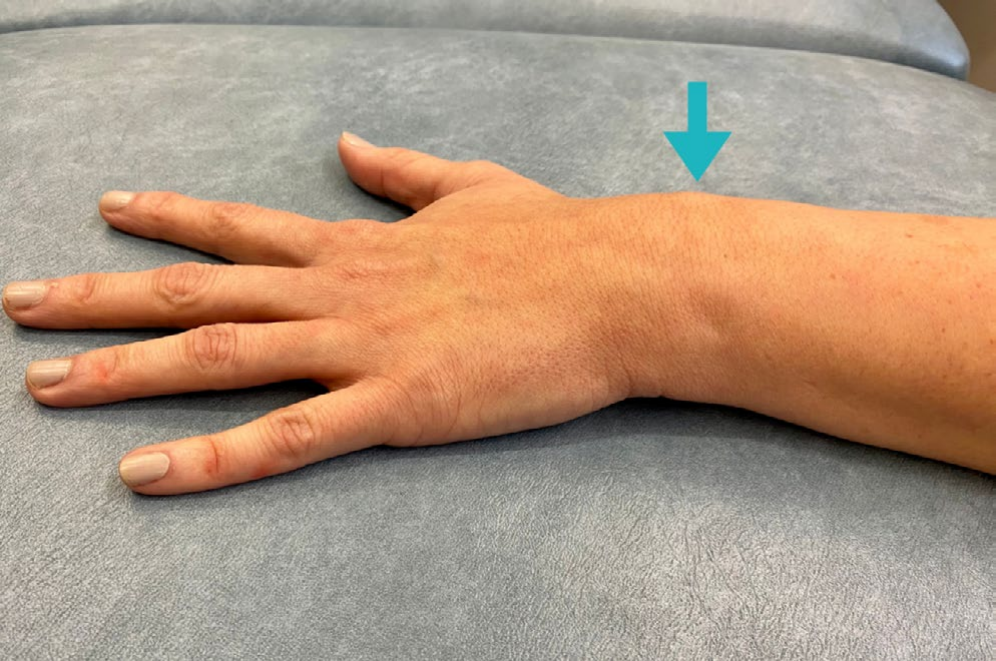
Raised contour at the proximal intersection. The blue arrow highlights the area of mild raised contour, mild swelling, on the dorsal aspect of the patient's left forearm, located approximately 4 cm proximal to the Lister's tubercle, at the proximal intersection.

Physical examination revealed mild raised contour, mild swelling, of the skin in the area of discomfort on the patient's left forearm, approximately 4 cm proximal to the Lister's tubercle, but there were no signs of skin discoloration or temperature differences compared to the contralateral hand (Figure 2). During palpation of the raised contour area, mild crepitus was felt under the skin, accompanied by moderate discomfort.

Symptoms were reproduced, and crepitus was noted during active, passive, and resisted range of motion, specifically during both extension of the first metacarpophalangeal joint (MCPJ) and abduction of the first carpometacarpal joint (CMCJ). However, the remainder of the assessment, including active, passive, and resisted range of motion of the elbow, forearm, wrist, and fingers, did not elicit significant symptoms. Overall, the range of motion and muscle strength in the wrist, forearm, and fingers were not impaired.

Further special tests were conducted to explore other possible differential diagnoses. The Finkelstein test, used to assess for De Quervain's tenosynovitis, was negative. Provocation tests for lateral and medial epicondylalgia also yielded negative results. Neurodynamic tests for radial, ulnar, and median nerves were negative, as were assessments of the shoulder girdle and cervical spine. No referred or radiating symptoms were provoked. Functional tests, which replicated her work‐related activities, such as lifting heavy objects with a wide grip, elicited the same pain she had described, clearly linking this action to her symptoms.

### 2.3 Sonographic Assessment

The sonographic assessment in this case was conducted using a high‐resolution linear transducer, allowing for clear delineation of superficial dorsal wrist anatomy, including tendon contours and compartmental interfaces. Our scanning protocol is supported by recent methodological insights provided by Corvino et al. (2024), who detailed an advanced approach to high‐frequency ultrasound of the quadrangular joint in carpal boss cases. Their discussion of probe positioning, optimization of focus and field‐of‐view, and the use of gel stand‐off pads reinforces the broader principles that underpin precise ultrasound imaging of dorsal wrist structures. Although their anatomical focus differs, the technical framework aligns conceptually with the imaging objectives in the present case and supports the rationale for dynamic, resolution‐enhanced sonography in detecting subtle tendon interactions.

#### 2.3.1 Evaluation of Surrounding Structures

Initial scanning of the contralateral asymptomatic limb established normal reference values. On the symptomatic side, the dorsal tendon compartments, distal flexors, and first compartment appeared normal with no evidence of tenosynovitis, sheath fluid, or retinacular thickening. Bone surfaces, hand and finger joints, and carpal tunnel structures were unremarkable. The extensor and flexor tendons maintained normal echogenicity and architecture. The radial, median, and ulnar nerves were traced from the axillary area to the wrist without signs of neuropathy or entrapment. No cysts, foreign bodies, or other space‐occupying lesions were identified.

#### 2.3.2 Proximal Intersection

**FIGURE 3:**
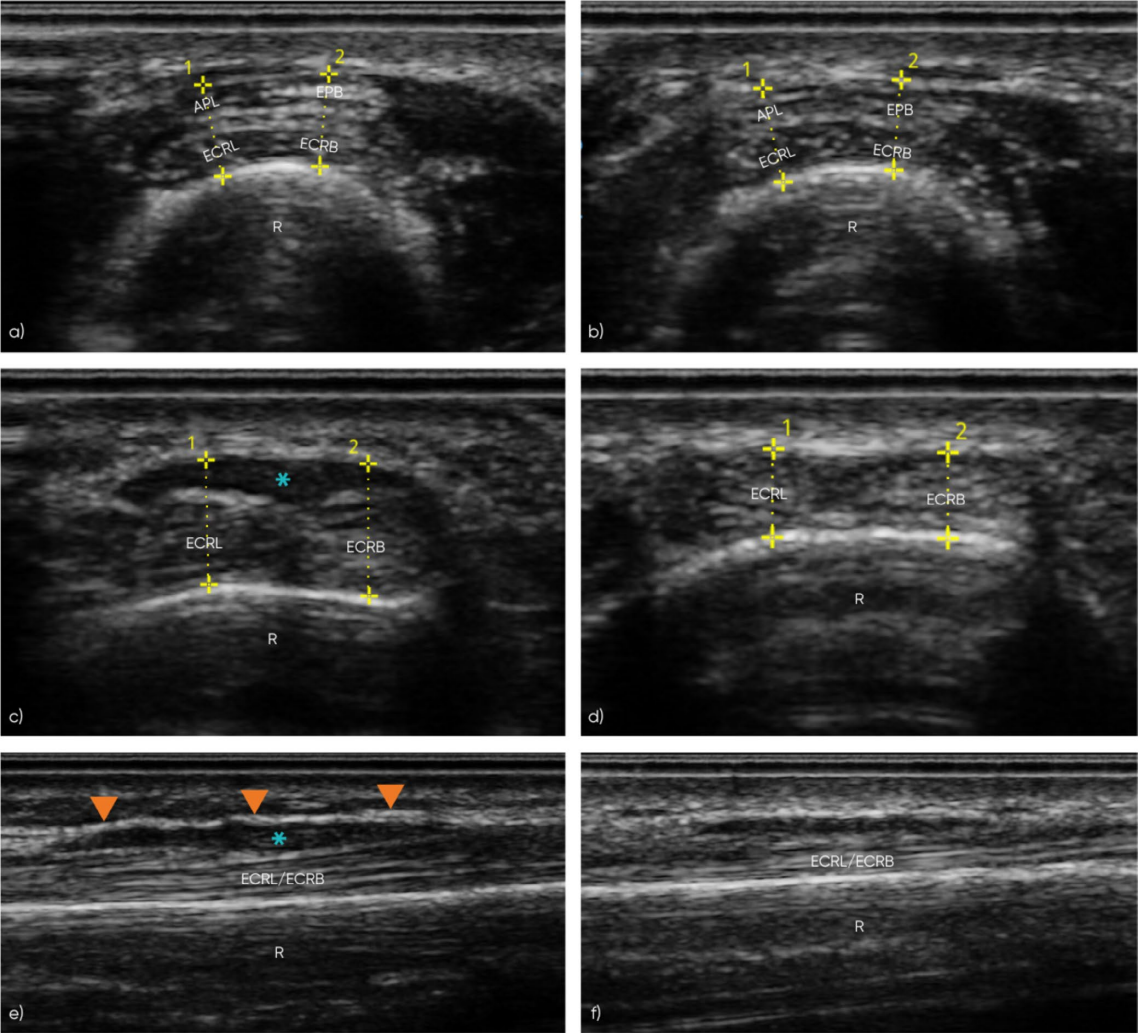
Ultrasound assessment of the symptomatic and asymptomatic limbs, at the proximal intersection, during the initial appointment. The figure displays short‐axis and long‐axis ultrasound images comparing the symptomatic (left column) and asymptomatic (right column) limbs. Short‐axis images at the proximal (a, b) and distal (c, d) sites of the proximal intersection highlight tendon morphology and sites of measurements, while long‐axis views (e, f) demonstrate EPB muscle characteristics. (a, b) Short‐axis images at the proximal site of the proximal intersection (ECRL‐APL and ECRB‐EPB). (a) Symptomatic limb shows normal tendon thickness and echogenicity. (b) Asymptomatic limb demonstrates similar normal findings. (c, d) Short‐axis images at the distal site of the proximal intersection (ECRL‐EPB and ECRB‐EPB). (c) Symptomatic limb shows mild tendon thickening and reduced echogenicity, consistent with tendinosis. (d) Asymptomatic limb displays normal tendon appearance and echogenicity. (e, f) Long‐axis views of the EPB muscle. (e) Symptomatic limb reveals a hypertrophied EPB muscle (blue asterisk) with thickened superficial connective tissue/fascia (orange arrowheads) and mild hypoechogenicity of the muscle, indicative of edema. (f) Asymptomatic limb shows normal EPB muscle morphology. *R*, radius; ECRL, extensor carpi radialis longus tendon; ECRB, extensor carpi radialis brevis tendon; APL, abductor pollicis longus tendon; EPB, extensor pollicis brevis tendon; blue asterisk, extensor pollicis brevis muscle; orange arrowheads, superficial connective tissue/fascia; yellow dotted lines with numbers, measurement sites for tendon thickness recorded during ultrasound scanning.

For the assessment of the proximal intersection, we followed the methodology of Christiaanse et al. (2014), obtaining measurements at two key sites in the short axis: the proximal site where the medial margins of the APL and ECRL align, and the distal site where the medial margins of the ECRL and EPB align. Long‐axis views were also captured for further evaluation. The obtained measurements are summarized in Table 1, while the corresponding ultrasound images are presented in Figure 3.

**TABLE 1:** Tendon thickness measurements at the proximal intersection in patient's asymptomatic and symptomatic limbs in millimeters (mm).

Site	Tendon pair	Asymptomatic (mm)	Symptomatic (mm)
Proximal site	ECRL‐APL	3.35	3.54
	ECRB‐EPB	3.17	3.51
Distal site	ECRL‐EPB	2.74	4.33
	ECRB‐EPB	2.67	4.6

Abbreviations: APL‐abductor pollicis longus; ECRB, extensor carpi radialis brevis; ECRL, extensor carpi radialis longus; EPB, extensor pollicis brevis.

In the asymptomatic limb, the proximal intersection exhibited a normal tendon appearance with measurements of 3.35 mm (ECRL‐APL) and 3.17 mm (ECRB‐EPB), at the proximal site. At the distal site, the measurements were 2.74 mm (ECRL‐EPB) and 2.67 mm (ECRB‐EPB). Long‐axis views confirmed the normal fibrillar appearance of the tendons. In the symptomatic limb, the proximal site showed normal tendon appearance and echogenicity, with measurements of 3.54 mm (ECRL‐APL) and 3.51 mm (ECRB‐EPB). However, at the distal site, mild thickening and loss of normal echogenicity were observed in both the ECRL and ECRB tendons, with measurements of 4.33 mm (ECRL‐EPB) and 4.6 mm (ECRB‐EPB). No anechoic fluid was observed in the tendon sheath nor Power Doppler imaging showed any evidence of hyperemia. Findings were indicative of tendinosis of the ECRL and ECRB tendons at the distal site of the proximal intersection.

The muscle belly of the EPB appeared slightly larger in cross‐section and more flattened in both short‐ and long‐axis views compared to the contralateral limb. Additionally, the superficial connective tissue fascia of the muscle appeared slightly more echogenic and thicker compared to the asymptomatic side, while the intra‐substance of the muscle itself was slightly more hypoechoic. Power Doppler imaging showed no evidence of hyperemia. Findings were indicative of overdeveloped, hypertrophied EPB muscle, and mild muscle oedema.

**FIGURE 4:**
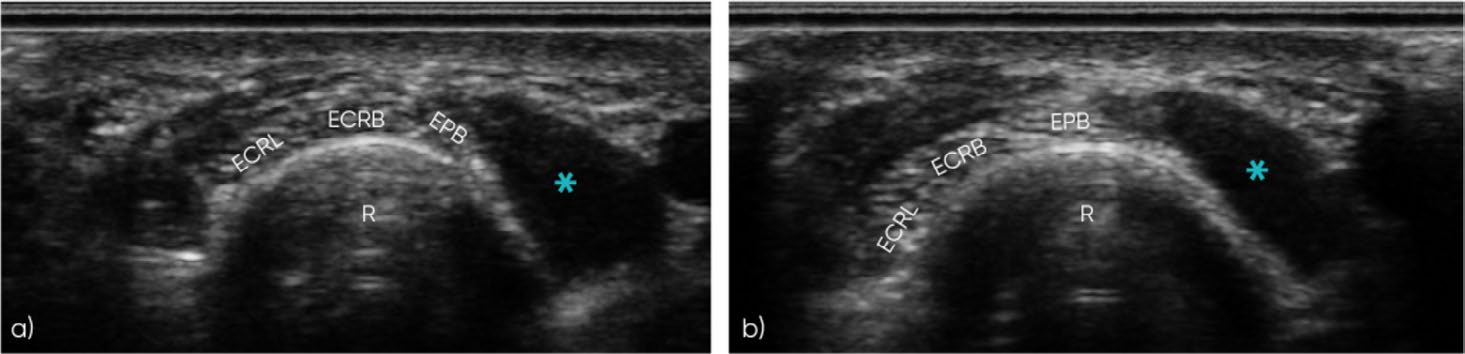
Dynamic ultrasound assessment of the extensor pollicis brevis muscle at the proximal intersection. (a, b) Short‐axis ultrasound images of the symptomatic limb. (a) At rest, the second compartment tendons (ECRL and ECRB) remain almost centrally aligned. (b) Contraction of the EPB muscle, during extension of the metacarpophalangeal joint, results in significant radial displacement of the second compartment tendons. *R*, radius; ECRL, extensor carpi radialis longus tendon; ECRB, extensor carpi radialis brevis tendon; EPB, extensor pollicis brevis tendon; blue asterisk, extensor pollicis brevis muscle.

Dynamic ultrasound assessment revealed that the EPB muscle, during contraction, pushed the second compartment tendons from their central position more radially (Figure 4). This displacement was most prominent during the extension of the first MCPJ, correlating with the pain elicited during this movement. In contrast, abduction of the first CMCJ caused only minimal radial displacement of the second compartment, which was not significant enough to reproduce the patient's symptoms.

### 2.4 Case Resolution

The patient underwent conservative management, beginning with immobilization using a cock‐up wrist and thumb spica splint in 15° extension for 3 weeks. She was instructed to wear the splint during hands‐on activities, remove it intermittently for wrist mobilization, and sleep with it on. A short course of ibuprofen (600 mg, three times daily for 7 days) was prescribed for pain control, consistent with literature recommendations for first‐line treatment with splinting and anti‐inflammatory medication (Schmidt et al. 2021). A longer course of medication was deemed unnecessary due to the patient's rapid responsiveness.

Activity modification was integral to the treatment plan, with her workload adjusted to reduce hands‐on activities while maintaining functionality through administrative tasks. Although complete rest is recommended in the literature (Schmidt et al. 2021), this was not feasible in her occupational setting. After 3 weeks, the patient transitioned to progressive rehabilitation, including mobilization, strengthening of the wrist and elbow flexors and extensors, and stretching exercises. This program extended to 8 weeks, exceeding the literature's recommended 4–6 weeks (Schmidt et al. 2021), resulting in full resolution of symptoms. The strengthening phase of the rehabilitation program focused on neuromuscular control, endurance, and gradual load tolerance, comprising low to moderate resistance with higher repetitions to avoid inducing further hypertrophy.

**FIGURE 5:**
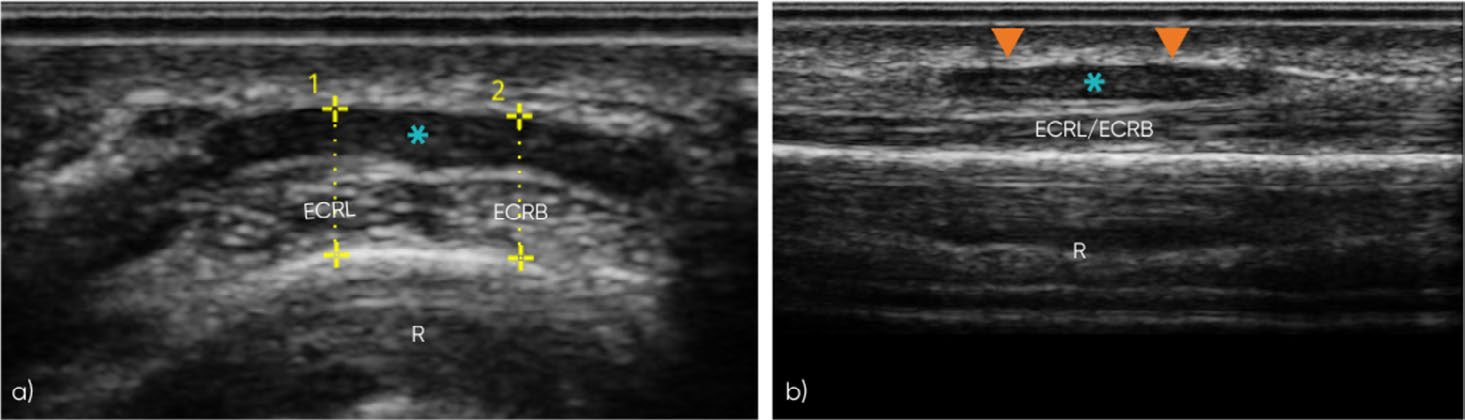
Ultrasound assessment of the symptomatic limb, at the proximal intersection, post‐treatment. (a) Short‐axis ultrasound image of the distal site of the proximal intersection demonstrating normal tendon echogenicity and thickness post‐treatment, with measurements of 3.83 mm (ECRL‐EPB) and 3.72 mm (ECRB‐EPB). No anechoic fluid or signs of hyperemia were detected. (b) Long‐axis ultrasound image of the EPB muscle at the same site demonstrating persistent hypertrophy of the muscle belly, which occupied considerable space above the second compartment. The muscle exhibited normal echogenicity, and the superficial connective tissue sheath showed comparable thickness to the contralateral limb, indicating no evidence of muscle edema. *R*, radius; ECRL, extensor carpi radialis longus tendon; ECRB, extensor carpi radialis brevis tendon; blue asterisk, extensor pollicis brevis muscle; orange arrowheads, superficial connective tissue/fascia; yellow dotted lines with numbers, measurement sites for tendon thickness recorded during ultrasound scanning.

While most cases of intersection syndrome respond to conservative management within 2–3 weeks (Schmidt et al. 2021), our patient required 11 weeks, likely due to continued occupational strain despite activity modification. At the end of this treatment program, ultrasound assessment of the symptomatic limb revealed that the distal site tendons displayed normal echogenicity and thickness, with measurements of 3.83 mm (ECRL‐EPB) and 3.72 mm (ECRB‐EPB). No anechoic fluid was observed within the tendon sheath, and Power Doppler imaging showed no evidence of hyperemia. The EPB muscle belly remained prominent, occupying considerable space above the second compartment, but exhibited normal echogenicity and a superficial sheath thickness comparable to the contralateral limb. These findings indicated persistent EPB muscle hypertrophy without evidence of muscle edema (Figure 5).

At the 6‐month follow‐up, the patient reported being completely symptom‐free, with no recurrence of pain, discomfort, or functional limitations in her daily activities.

## 3 Discussion

Intersection syndrome was first described by Velpeau in 1841, who identified tenosynovitis affecting the forearm's extensor tendons (Balakatounis et al. 2017). The term *intersection syndrome*, however, was introduced by Dobyns et al. in 1978, delineating the condition as a distinct clinical entity marked by inflammation at the intersection of the first and second dorsal compartments (Dobyns et al. 1978). Throughout its history, alternative nomenclatures such as *peritendinitis crepitans*, *crossover syndrome, adventitial bursitis, subcutaneous perimyositis with APL syndrome*, *and bugaboo forearm* have been employed, emphasizing the clinical presentation, primarily crepitus and localized pain, characteristic of the condition (Balakatounis et al. 2017).

### 3.1 Clinical Features and Risk Factors

PIS typically presents with pain, swelling, crepitus, and sometimes erythema localized to the dorsal‐radial forearm, approximately 4–8 cm proximal to the radial styloid (Tan et al. 2024; Schmidt et al. 2021; Montechiarello et al. 2010). In our case, the patient exhibited pain and crepitus but without overt erythema. Mild skin contour elevation was observed, which may reflect underlying EPB muscle hypertrophy visualized on ultrasound, rather than a true inflammatory swelling.

PIS has been predominantly described in athletes, particularly those involved in repetitive wrist‐intensive sports such as rowing, paddling, and weightlifting (Murphy et al. 2025). It has also been documented in other activities, including tennis, skiing, sailing, and even weight training (Hoy et al. 2019). The condition often manifests shortly after an increase in training volume or initiation of a new activity (Montechiarello et al. 2010). Although primarily associated with athletic activities, the syndrome can also occur in occupational or recreational settings involving similar repetitive motions (Schmidt et al. 2021; Christiaanse et al. 2014). While its overall prevalence in the general population is relatively low, with reported incidence rates ranging from 0.2% to 0.37% (Draghi and Bortolotto 2014), PIS remains an important differential diagnosis for dorsal radial forearm pain associated with overuse. Previous studies have shown that PIS can occur in occupational settings. In a four‐year prospective study, Pantukosit et al. (2001) found that 0.37% of patients presenting with wrist pain had PIS, the majority of which were related to occupational activities most commonly linked to repetitive manual labor in agricultural settings. Their findings specifically implicate twisting, pulling, and radial deviation tasks such as rice harvesting as contributing mechanisms. Similarly, Descatha et al. (2008) further reported a case of PIS in a supermarket cashier following a lifting‐related wrist strain, supporting the notion that sustained or repetitive occupational loading, even without athletic intensity, can trigger the syndrome. In our case, the patient was a nursery practitioner performing daily lifting of toddlers and equipment, involving sustained first MCPJ extension with a wide grip. Notably, her symptoms developed 4–5 weeks after starting the job, a timeline similar to that observed in sport‐related PIS when novel or intensified wrist demands are introduced (Montechiarello et al. 2010).

Traditionally, the mechanism of this overuse injury has been attributed to repetitive wrist flexion‐extension or radial deviation movements, which generate friction between the first and second dorsal compartments (Tan et al. 2024; Schmidt et al. 2021; Hoy et al. 2019; Montechiarello et al. 2010; Christiaanse et al. 2014). While such movements remain central, our case challenges this narrow view. Provocative testing and dynamic ultrasound demonstrated that the EPB muscle belly displaced the second compartment tendons radially during MCPJ extension, suggesting that the root cause was not wrist motion per se, but rather mechanical crowding at the intersection site. These findings underscore the importance of task‐specific analysis and support the use of real‐time sonography to detect tendon displacement and compartmental interaction that static assessment may overlook.

### 3.2 Diagnosis

The diagnosis of PIS is primarily clinical and based on a thorough patient history and physical examination (Murphy et al. 2025; Draghi and Bortolotto 2014). In fact, studies suggest that up to 70% of cases can be identified accurately through careful history‐taking and clinical evaluation alone, making imaging supplementary rather than essential in straightforward presentations (Balakatounis et al. 2017). In cases, though, where the clinical diagnosis is uncertain or atypical, the syndrome may be diagnosed on magnetic resonance imaging (MRI), which shows high signal intensity on T2‐weighted sequences within the tendons and peritendinous fluid (Christiaanse et al. 2014). The syndrome can also be diagnosed using ultrasound imaging, which serves as a valuable diagnostic adjunct due to its cost‐effectiveness, high contrast resolution for superficial structures, and the ability to perform dynamic assessments (Malliaropoulos and Daoukas 2024; Alghamdi et al. 2024).

The diagnostic approach in our case aligns with recent findings by Corvino et al. (2023), who employed high‐resolution dynamic ultrasound to diagnose de Quervain's tenosynovitis in a non‐athletic caregiver, a case they termed “Daddy wrist.” Their report demonstrated the importance of dynamic sonography in detecting functional stress‐related changes, such as tendon edema, retinacular thickening, and subcompartmentalization within the first dorsal compartment. Similarly, our case illustrates how real‐time ultrasound can reveal subtle, motion‐induced tendon interactions; in this instance, displacement of second compartment tendons by a hypertrophied EPB muscle during MCPJ extension. Both cases reinforce the value of dynamic imaging in evaluating overuse syndromes that may not be apparent on static scans, especially in occupational or caregiving contexts where atypical biomechanical patterns are present.

#### 3.2.1 Ultrasound Imaging

The primary utility of ultrasound lies in confirming the diagnosis and ruling out alternative conditions often included in the differential diagnoses, such as De Quervain's tenosynovitis, fractures, osteoarthritis, or anatomical variants (Draghi and Bortolotto 2014). Ultrasound findings typically include tendon thickening, peritendinous edema, fluid accumulation within tendon sheaths, and hypervascularity on Power Doppler at the intersection of the first and second compartments (Hoy et al. 2019). Additionally, disruption of the hyperechoic plane separating the two tendon groups, as well as signs of tendinosis or subcutaneous edema, can further support the diagnosis (Tan et al. 2024; Montechiarello et al. 2010).

To our knowledge, the only study that has reported normal tendon thickness figures at the proximal intersection is by Christiaanse et al. (2014). They proposed that the combined thickness of the superimposed tendons at the proximal intersection should not exceed 4 mm (3–4 mm) in women and 4.8 mm (3.7–4.8 mm) in men. They also recommended comparison with the contralateral asymptomatic side, since thicknesses should be similar between sides. Additionally, they suggest comparing measurements at proximal and distal sites of the intersection, as these should also show comparable values.

Hypertrophy of the first compartment muscle has been previously described in the literature, particularly in athletes, where it has been identified as a key pathological mechanism contributing to PIS (Hoy et al. 2019). In those cases, surgical intervention, considered the gold standard, allowed direct visualization and confirmation of muscle hypertrophy and its role in the pathology.

In our case, the patient presented with asymmetry in the first compartment muscle belly, which was barely visible on the asymptomatic side but appeared larger on the symptomatic side. Sonographic findings of mild focal hypoechogenicity, thickening, and increased hyperechogenicity of the superficial fascia suggested muscle edema. Post‐treatment ultrasound revealed a reduction in muscle flattening and normal echogenicity, supporting the hypothesis that mechanical friction during occupational tasks, such as repetitive gripping and prolonged extension of the first MCPJ, contributed to localized edema secondary to hypertrophy.

## 4 Conclusion

This case report contributes ultrasound‐based evidence identifying EPB muscle belly hypertrophy, combined with associated edema and dynamic tendon displacement, as a potential pathomechanism in an occupational presentation of PIS. While occupational cases have previously been described, this report adds novel insight into dynamic mechanical contributors visualized in real time. Based on dynamic ultrasound findings, we propose that lifting activities involving prolonged extension of the first MCPJ with a wide grip may induce mechanical friction and stress between the first and second dorsal compartments, potentially leading to PIS. These findings expand the understanding of PIS pathophysiology, highlighting an alternative mechanism that warrants further investigation. Future studies should focus on exploring dynamic interactions between compartmental tendons and muscles during occupational or repetitive tasks. Additionally, assessing the role of muscle hypertrophy and edema in early or atypical presentations of PIS could improve diagnostic precision and inform tailored intervention strategies.

## Conflicts of Interest

The authors declare no conflicts of interest.

## Data Availability

The data that support the findings of this study are available on request from the corresponding author. The data are not publicly available due to privacy or ethical restrictions.
